# Tolerance to Plant Pathogens: Theory and Experimental Evidence

**DOI:** 10.3390/ijms19030810

**Published:** 2018-03-11

**Authors:** Israel Pagán, Fernando García-Arenal

**Affiliations:** Centro de Biotecnología y Genómica de Plantas (UPM-INIA) and E.T.S. Ingeniería Agronómica, Alimentaria y de Biosistemas, Universidad Politécnica de Madrid, 28223 Madrid, Spain

**Keywords:** plant defense responses, tolerance to plant pathogens, range and point tolerance, tolerance mechanisms to pathogens

## Abstract

The two major mechanisms of plant defense against pathogens are resistance (the host’s ability to limit pathogen multiplication) and tolerance (the host’s ability to reduce the effect of infection on its fitness regardless of the level of pathogen multiplication). There is abundant literature on virtually every aspect of plant resistance to pathogens. Although tolerance to plant pathogens is comparatively less understood, studies on this plant defense strategy have led to major insights into its evolution, mechanistic basis and genetic determinants. This review aims at summarizing current theories and experimental evidence on the evolutionary causes and consequences of plant tolerance to pathogens, as well as the existing knowledge on the genetic determinants and mechanisms of tolerance. Our review reveals that (i) in plant-pathogen systems, resistance and tolerance generally coexist, i.e., are not mutually exclusive; (ii) evidence of tolerance polymorphisms is abundant regardless of the pathogen considered; (iii) tolerance is an efficient strategy to reduce the damage on the infected host; and (iv) there is no evidence that tolerance results in increased pathogen multiplication. Taken together, the work discussed in this review indicates that tolerance may be as important as resistance in determining the dynamics of plant-pathogen interactions. Several aspects of plant tolerance to pathogens that still remain unclear and which should be explored in the future, are also outlined.

## 1. Introduction

Parasites are an important fraction of living organisms, and by some estimates they constitute over half the organisms on Earth [1]. This means that, along their life span, hosts will be recurrently challenged by parasites, and plants are not an exception. Plant parasites may be pathogens, thus causing diseases that have a negative impact in the fitness of the infected hosts [2,3,4]. As a consequence, plant pathogens are important ecological agents that may affect the composition of plant populations [5,6] and in extreme cases, cause the local extinction of host species [7]. In addition, plant pathogens are responsible for important yield reductions in crops. It has been estimated that between 13% and 16% of crop production worldwide is lost directly (yield, quality, etc.) every year due to pathogens [8,9]. The FAO estimates that indirect losses (effect, rural communities, environment, etc.) may increase these numbers up to 20–40% [10], with subsequent economic and social impacts [8,9].

To cope with pathogens, hosts have developed a variety of defense mechanisms to avoid/limit infection and its negative effects [11]. The two main mechanisms of plant defense against pathogens are resistance, i.e., the host’s ability to limit pathogen multiplication [12,13], and tolerance, i.e., the host’s ability to reduce the effect of infection on its fitness regardless of the level of pathogen multiplication [14,15]. They represent two fundamentally different strategies to deal with pathogens: resistance reduces the risk of infection and/or the replication rate of the pathogen in the host, whereas tolerance does not. Although both resistance and tolerance can impose selection on the pathogen [16,17,18], these two types of defenses may lead to different ecological and evolutionary interactions between plants and pathogens [15,19]. For instance, it is predicted that if plants evolve resistance, this would reduce the prevalence of the pathogen in the host population, whereas if plants evolve tolerance, prevalence will increase [16]. Thus, both resistance and tolerance may have a significant, but different, impact on the dynamics of plant and pathogen populations. In the past decades, plant researchers have devoted considerable effort to understand the molecular basis and evolutionary consequences of both defense strategies. While there is extensive literature on resistance to plant pathogens, tolerance has received comparatively less attention. However, studies on plant tolerance have led to major insights into its evolution and mechanistic basis, mostly in the past three decades.

The concept of tolerance first appeared in the literature more than a century ago [20] and was initially defined in the crop science field as the ability to suffer little loss of plant yield (i.e., grain production) upon pathogen infection [21]. Although experimental evidence for tolerance in crops remains elusive [22], this definition of tolerance continues to be useful in the context of crops and is related to plant fitness, as higher symptom severity and decreased fitness are often correlated [23,24]. In 1995, Fineblum & Rausher [25] published a seminal paper in which the authors demonstrated the existence of a trade-off between resistance and tolerance of *Ipomea purpurea* to herbivory. Based on this trade-off, they constructed a mathematical model for the evolution of both defense mechanisms that predicted a mutual constraint for the evolution of tolerance and resistance in wild plant populations. Subsequently, tolerance became a prevalent concept in plant evolutionary ecology, initially in the context of plant herbivory but soon after expanded to plant-pathogen interactions [14,26]. As a consequence, hypotheses concerning the joint evolution of tolerance and resistance have been both theoretically and experimentally examined, and interesting debates regarding the terminology and the quantification of tolerance have been held.

Here, we review first the theory and subsequent models on the evolution of tolerance that can be applied to plant-pathogen interactions, as well as the experimental analyses of theory predictions, and then the current knowledge on the mechanisms of tolerance. We focus the review on tolerance to fungi and oomycetes, viruses, bacteria and parasitic plants. We do not consider phytopathogenic plant nematodes because, although tolerance to nematodes has been analyzed in an agronomic context, it has not been addressed from an evolutionary ecology perspective. We do not aim at an exhaustive review of the literature, but rather to provide an overview of the state-of-the-art in a topic of the evolution of plant-pathogen interactions that is receiving increasing attention by researchers.

## 2. Theory on the Evolution of Tolerance to Pathogens

The importance of pathogens to agriculture and human health [4,27], and the increasing awareness of the role of pathogens in structuring natural communities [28,29], has resulted in a well-developed theory on the evolution of tolerance to pathogen infection.

A first group of studies dealt with the conditions that result in the evolution of tolerance in the host. Early studies focused on tolerance to herbivory, but their assumptions and conclusions can easily be extended to tolerance to pathogens. Under the assumption that resources are limited and can be diverted into resistance or tolerance, but not into both, early models on the evolution of tolerance were constructed considering that resistance and tolerance were mutually exclusive. Based on these assumptions, van der Meijden et al. [30] proposed that the evolution of tolerance or resistance to plant damage would depend on the amount of nutrients available and on the plant growth rate. Herms & Mattson [31] translated this theory into a mathematical model, which predicted that tolerance would be favored in environments with high resource availability, as there would be no limitation of the amount of nutrient uptake needed to compensate losses to herbivory. On the other hand, when resources are limited, resistance would be a more advantageous strategy because it would prevent the loss of the few resources available. In addition, plants with faster growth rates and shorter lifespans would evolve resistance, as the amount of resources lost would be comparatively smaller than that of plants with slower growth rates and longer lifespans for which evolving tolerance would be more advantageous. Soon after this theory/model was developed, it was proposed that it could be generalized to pathogens, including plant pathogens [32]. Models soon incorporated the idea that resistance and tolerance might not be fully exchangeable if, for instance, tolerance evolved in response to stresses other than herbivory or disease, and/or resistance and tolerance were not genetically linked [33]. Under these assumptions, Mauricio et al. [34] built a model which predicted that both tolerance and resistance would coexist in the same host if costs and benefits of developing each defense strategy were non-additive, although host fitness was maximized only at maximum tolerance or maximum resistance. This model also contemplated that maximum tolerance resulted in over-compensation (i.e., infected plants have higher fitness than uninfected individuals). Similar predictions were made by Boots & Bowers [35], who incorporated different types of resistance in their model. Fornoni et al. [36] and Restif & Koella [19,37] went a step further into making models more realistic by introducing variable costs and benefits of tolerance and resistance, as well as nonlinear cost-benefit functions for both defense traits. These models predicted a fitness maximum at intermediate levels of tolerance and resistance when the benefit and cost functions are nonlinear. Another key conclusion of Restif & Koella [19,37] was that pathogens with low virulence could be more likely to select for their hosts’ tolerance, whereas high virulence could favor resistance. Based on the [19] model, more recent ones have shown that the type of parasitism may also affect the evolution of resistance or tolerance. For instance, infection by a sterilizing pathogen, i.e., one that drains the reproductive resources of the infected host, eventually leading to castration [38], promotes tolerance rather than resistance as far as the pathogen is not vertically transmitted [39]. Selection for tolerance rather than resistance would be stronger against sterilizing pathogens with high multiplication rates because the cost of limiting pathogen multiplication (resistance) tends to infinity for pathogens with higher multiplication rates [36], a prediction also derived from the Restif & Koella [19,37] models.

If hosts evolve tolerance, two main questions arise: (i) how is tolerances maintained in the host population? and (ii) what would be the consequences of host tolerance for pathogen evolution? In other words, how does tolerance affect host-pathogen co-evolution? Roy & Kirchner [16] built a simple model predicting that as a gene conferring tolerance to pathogens spreads in the host population, disease prevalence rises, increasing the advantage of carrying the tolerance gene. Therefore, under sustained pathogen infection pressure, any tolerance gene that can invade a host population will tend to be fixed by selection, provided that benefits of tolerance outweigh the costs, and genetic polymorphisms for tolerance would not be maintained in the host population. By contrast, a resistance gene reduces disease prevalence as it spreads in the host population, decreasing the advantage of carrying the resistance genes and polymorphisms for resistance, could be maintained by frequency-dependent selection [35,40,41,42]. Assuming that the two defense strategies were independent (unlinked genes), Restif & Koella [19] also predicted the absence of polymorphisms for host tolerance. Miller et al. [43] expanded previous models incorporating fitness costs of plant defenses in both host fecundity and mortality and considering pathogens with a free-living stage, but even so, their model predicted the fixation of tolerance (but not of resistance) in the host population. However, Best et al. [44] found that tolerance to the effects of disease-induced mortality (i.e., mortality tolerance), but not tolerance to the effect of the disease-induced reductions in fecundity (i.e., fecundity tolerance), led to polymorphic host populations. This was because mortality tolerance had a positive effect on pathogen fitness as it affects the infectious period, whereas fecundity tolerance was neutral, and the host population could be polymorphic for it. In the same sense, Best et al. [39], who examined the evolution of host defense to the sterilizing effects of parasitism, suggested that fecundity tolerance to sterilizing pathogens would result in genetic variation for this trait, although these authors do not explicitly address this possibility.

As it spreads through the host population, tolerance might also affect pathogen evolution. The Roy & Kirchner [16] model predicted that tolerance leads to increased prevalence of the pathogen in the host population. Restif & Koella [37] investigated if tolerance might have an impact on the evolution of other pathogen traits using a model, assuming that the host controlled pathogen virulence and that virulence and within-host multiplication were positively correlated [45]. This model predicted that a host-pathogen interaction would reach an evolutionary stable state at intermediate levels of host tolerance and pathogen multiplication. Higher or lower levels of pathogen multiplication would break this evolutionary stable state: High levels of pathogen multiplication would result in host extinction, whereas low levels of pathogen multiplication would result in the invasion of the host population by a more fecund genotype. This would lead to the pathogen extinction if the invading host genotype were more resistant than the resident tolerant one [37]. Miller et al. [43,46] showed that pathogens might evolve either higher or lower within-host multiplication rates depending on the nature of the tolerance mechanism: If tolerance reduced virulence by a constant factor, selection would always result in increased pathogen multiplication. Alternatively, if tolerance reduced virulence nonlinearly, being less effective against pathogens with higher multiplication rates, selection for faster or slower replicating pathogens would occur depending on higher or lower transmission rates, respectively. Similarly, van der Bosch et al. [18,47] found that increased tolerance selected for higher within-host pathogen titers. Importantly, these models quantified tolerance either as mortality tolerance or fecundity tolerance. As mentioned earlier, the Best et al. [44] model stressed that there was a crucial distinction between fecundity and mortality tolerance, with mortality (but not fecundity) tolerance having a positive effect on pathogen fitness (increased transmission). However, the authors noted two exceptions to this conclusion: (i) it excluded vertically transmitted pathogens, and (ii) fecundity tolerance may have an effect on pathogen fitness if it comes at the cost of a reduced host life span (i.e., shorter infectious period).

Finally, a third group of models aimed to understand how host- and pathogen-specific characteristics or environmental factors affect the evolution of tolerance. Kirchner & Roy [48] modeled the effect of host-pathogen specificity on defense traits. They showed that if the pathogen has low host genotype specificity, host genotypes with higher reproduction rates or longer lifespans, more likely to evolve tolerance [31], will over-compete short-lived host genotypes. Conversely, at higher host-pathogen specificity, long-lived genotypes have no advantage, and pathogen-mediated selection will favor polymorphisms in host resistance. Detilleux [49] simulated the effect of mortality tolerance on pathogen prevalence in host populations under human management. At odds with Roy and Kirchner [16], he found that high frequency of tolerant host genotypes resulted in intermediate pathogen prevalence. This result was attributed to the fact that, in managed host populations, non-tolerant individuals are maintained (no culling). Therefore, the infectious period is similar in tolerant and non-tolerant hosts, such that mortality tolerance does not result in increased risk of exposure to the pathogen. Although this model was built using an animal-pathogen system, it can be easily applied to crops. The effect of the spatial structure of the host population on the evolution of tolerance has also been modeled, predicting that a spatial structure slows the rate of invasion of tolerance in the host population, invasion being possible only if tolerance has a lower cost than resistance [50,51]. Lastly, Cousineau & Alizon [52] modeled the effect of sexual dimorphism on the evolution of host defenses. Their model predicted that, at an evolutionary stable state, pathogen virulence would be higher if there is sexual dimorphism for tolerance, as opposed to the absence of it. This is because in the model, sexual dimorphism allows the existence of super-tolerant host genotypes in one of the sexes, such that the cost of higher pathogen virulence is reduced.

All this theoretical work illustrates the complex effects that tolerance may have on host-pathogen co-evolutionary dynamics and how such effects may differ according to the way in which tolerance is understood (fecundity tolerance vs. mortality tolerance). Thus, experimentally testing the predictions of theory may be challenging and further depends on two critical questions: (i) how is tolerance defined? and (ii) what variable can be used to quantify tolerance? [14,44,53].

## 3. Measuring Tolerance to Pathogens

**Figure 1:**
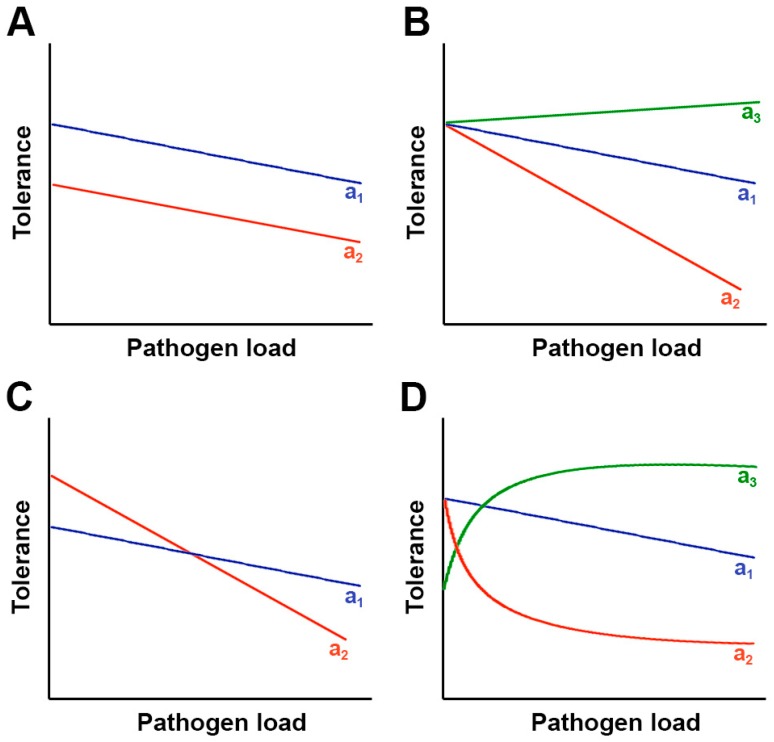
Range versus point tolerance. Depending on the scenario, range tolerance and point tolerance do not necessarily lead to the same conclusion. (**A**) Host genotype a_1_ (blue) has higher fitness than genotype a_2_ (red) when uninfected and at every pathogen load, but range tolerance is the same in both genotypes. Point tolerance will always indicate higher tolerance of genotype a_1_; (**B**) Host genotype a_1_ and a_2_ have the same fitness when uninfected, but range tolerance is higher in a_1_ than in a_2_. Point tolerance will agree with range tolerance at every pathogen load. Genotype a_3_ (green) overcompensates detrimental effects of infection at every pathogen load (positive slope of the reaction norm); (**C**) Host genotype a_1_ has lower fitness than genotype a_2_ when uninfected, but range tolerance is higher for genotype a_1_ than for a_2_. Point tolerance will agree with range tolerance at higher, but not at lower, pathogen load; (**D**) Both genotypes have the same fitness when uninfected. Range tolerance is linear for genotype a_1_ but not for genotype a_2_. Genotype a_2_ has lower range tolerance than a_1_ in the exponential part, but higher in the plateau, of the reaction norm. Point tolerance will always be higher for genotypes a_1_. Genotype a_3_ overcompensates the detrimental effect of pathogen infection up to a maximum.

We have previously defined tolerance as the host’s ability to reduce the effect of infection on its fitness regardless of the level of pathogen multiplication. That is, tolerance represents the ability of a host to cope with pathogen infection across a range of pathogen loads. The variation in a given trait across environments is known as the *reaction norm* [54]. Following the formal analysis of the reaction norm, tolerance is often represented by the slope of a regression of host fitness against pathogen load (Figure 1); the steeper the slope, the lower the tolerance [14,15,55,56]. If defined as a slope, tolerance is known as range tolerance, which cannot be measured on a single plant but across individuals of a given host type. Thus, to understand the genetic variation of range tolerance, it is necessary to measure fitness of genetically distinct groups of hosts (genotypes, populations, etc.) under different pathogen loads and to compare the slopes among these genetic groups. This approach was originally developed to analyze tolerance of plants to herbivores [26] and has also been applied to pathogens (Table 1). In addition to being measured as a slope, tolerance can also be measured at one single pathogen load, that is, at a single point on the slope of the reaction norm, which is known as point tolerance [14,57] (Table 1). We would like to stress the difference between range and point tolerance, as conclusions from these two measures of tolerance might be not always the same [14]. Given that reaction norms for two hosts may intersect and that reaction norms may be non-linear, conclusions derived from the comparison of point tolerances would depend upon the point in the reaction norm at which the measurement is made (Figure 1). Although several works have analyzed point tolerance (e.g., [58,59]), range tolerance is generally regarded as a more accurate approach for the reasons stated above. Indeed, statistical approaches based on the analysis of random regressions have been proposed as the most suitable for the analysis of tolerance [60,61]. This proposition has not been confirmed as, to our knowledge there are no analyses in plant-pathogen interactions comparing point and range tolerance (Table 1).

Studies on tolerance to plant pathogens utilize plant fitness as the dependent variable [55,56]. From an evolutionary standpoint, the fitness of a genotype is defined as the expected number of offspring contribution of an individual of such genotype to the next generation [62]. However, the key variables for measuring fitness may vary depending on the context, the question addressed, and the experimental constraints of each particular study [63,64]. For instance, pathogen infection may affect plant fecundity directly or through reducing survival. In plants with short generation times, such as *Arabidopsis thaliana*, lifespan may not be long enough for a detectable effect of infection on survival, and fecundity (i.e., number of offspring produced, which in plants is generally quantified as the number of viable seeds) may be enough to measure tolerance ([59,65,66], but see [67]). On the other hand, in long-lived plants, the effect of infection on lifespan may have large effects on progeny production, and both fecundity and mortality should be measured to calculate host fitness. Most experimental studies on the evolution of virulence used plant fecundity upon infection as a measure of tolerance, perhaps because most host species used in these studies have short lifespans (Table 1; but see [68,69]). However, tolerance can also be quantified as reduced plant mortality. Plant mortality can be quantified in different ways: (i) Case mortality (i.e., the probability of pathogen-induced host death once infected)—larger values represent higher virulence; (ii) expected time until death from infection—smaller values represent higher virulence; and (iii) lethal dose—smaller values represent higher virulence [63]. Both case mortality [70] and expected time until death from infection [66,67] have been used to analyze plant tolerance to pathogens. Case mortality is a good choice as it can be used to test models that include or do not include pathogen within-host dynamics [63]. However, this measure only provides information on the number/frequency of infection-associated deaths, which is a rather qualitative measure of host fitness. On the other hand, the expected time to death provides a more quantitative description of the reduction in host fitness due to mortality [63]. These two measures do not necessarily cross-correlate [63]. Thus, even when mortality tolerance is used in two studies, comparison between values may be difficult. Finally, the question to be answered may determine the best measure of tolerance. As previously mentioned, if one is interested in analyzing the effect of plant tolerance on the host population dynamics, both mortality and fecundity will be informative parameters. On the other hand, to address the effect of tolerance on strict horizontally transmitted pathogen fitness, it would be more informative to use mortality tolerance: the key trait for pathogen fitness is transmission, which is positively associated with the infectious period, and the infectious period depends on the infection-induced mortality. Note that this distinction would not prevail for pathogens both horizontally and vertically transmitted, as in this case, plant progeny is also a component of pathogen fitness.

To interpret the studies discussed below, the reader should keep in mind that in most of them, tolerance was measured as range tolerance, and plant fecundity was the relevant plant trait (Table 1). Cases in which this is not so are specified.

## 4. Experimental Evidence of Tolerance in Plant-Pathogen Interactions

Although the evolution of plant tolerance has mainly been analyzed in the context of tissue damage by herbivores [26], a significant body of experimental work has dealt with the evolution of tolerance to plant pathogens.

### 4.1. Fungi and Oomycetes

Tolerance of plants to fungi and oomycetes has received considerable attention, and studies almost always define tolerance as a reduced effect of infection on plant fecundity. There is a considerable literature on tolerance of crops to fungi (e.g., [71,72,73]), although few tolerant genotypes against fungi have been deployed in the field [73].

The evolution of tolerance to fungi in wild plants has received also considerable attention. Indeed, in 2000, Roy and Kirchner cited a dozen of works on wild plant–rust interactions that conform to their predictions of low levels of variation for tolerance (Table 1). At that time, evidence of genetic diversity in tolerance to fungi other than rusts had been also reported [56,74]. These studies found evidence of tolerance costs, and proposed that costs would be explained by a linked evolution of tolerance and resistance. Interestingly, in agreement with the Mauricio et al. [34] model, the cost of tolerance was linear. Further experimental work in perennial shrub communities showed that despite high prevalence of fungal diseases, reduction of plant survival and flowering was minimal [75], which suggested that plant-native fungus interactions would result in higher tolerance than interactions with introduced fungi. In other words, that pathogen’s host specificity would favor the evolution of tolerance. This hypothesis, formally modeled by Kirchner & Roy [48] as summarized above, was experimentally tested by Inglese & Paul [76], who analyzed tolerance of *Senecio vulgaris* to the native fungus *Coleosporium tussilginis* and the introduced *Puccinia lagenophorae*. In agreement with theoretical predictions, tolerance was higher to the native than to the introduced fungus. As could be expected, studies on the evolution of tolerance to fungi in *Arabidopsis thaliana* also exist. We would like to highlight the series of works by Salvaudon & Shykoff on the interaction between *A. thaliana* and the oomycete *Hyaloperonospora arabidopsidis* in which genetic variation for tolerance was found in the plant [77,78,79]. Interestingly, tolerance depended on the amount of resources available [77] as predicted by Herms & Mattson [31] and on the plant lifespan and reproduction rate [78] as predicted by Kirchner and Roy [48].

### 4.2. Viruses

Tolerance to virus infection has been widely analyzed in crops. For example, both point and range tolerance has been described for *Tomato yellow leaf curl virus* (TYLCV) in tomato [80,81], to *Barley yellow dwarf virus* (BYDV) in barley and oat [82,83,84], or to potyviruses such as *Blackeye cowpea mosaic virus* (BICMV) in cowpea [85], among others. Because the objective of these works was selecting plant lines for crop improvement, they measured tolerance as the host ability to maintain grain/fruit yield upon virus infection, and only during one growing season. The main conclusion that can be extracted from these studies is that tolerance is not uncommon in crops and seems to have evolved in phylogenetically distant crops. However, these studies yield limited information on the evolution of tolerance. A notable exception is the series of studies by Desbiez and Lecoq, who reported that the deployment in Martinique of zucchini genotypes tolerant to *Zuchinni yellow mosaic virus* (ZYMV) infection resulted in the appearance of more virulent ZYMV strains with an associated fitness penalty in non-tolerant hosts [86,87,88]. These studies have the additional interest of referring to the only instance, to our knowledge, in which tolerance has been bred into crops and deployed for the control of a relevant viral disease.

Most analyses of tolerance to viruses with an evolutionary perspective have used the *Cucumber mosaic virus* (CMV) as a model. In separate studies, Carr et al., analyzed point [58] and range [89] tolerance of *Mimulus gutattus* to CMV, finding polymorphisms for fecundity tolerance, but the level of genetic variation for this trait was very low [58,89]. This observation was consistent with the Roy and Kirchner [16] model, which predicted no or low polymorphisms for tolerance. Interestingly, no cost for *M. gutattus* tolerance to CMV was detected, a central assumption of the Roy and Kirchner’s model, but not of other models predicting genetic polymorphisms for tolerance (see above). Carr et al. [58,89] quantified tolerance as the effect of infection on progeny production (Table 1). Thus, their results did not support the predictions made by the Best et al. [44] model of genetic variation for tolerance if this trait was measured as fecundity tolerance. At odds, Pagán et al. [65,66] found a large variation for fecundity tolerance of *A. thaliana* to CMV, with medium to high heritability. A possible explanation for this discrepancy between analyses in both hosts is that fecundity tolerance to CMV has an unexplored cost for *M. guttatus*. CMV is seed transmitted in *A. thaliana*, which would invalidate the argument of Best et al. [44], as host fecundity would have an effect on pathogen fitness. Thus, polymorphisms for tolerance may arise even when the relevant host trait is under apparent pathogen-mediated selection. Further analyses of *A. thaliana* tolerance to infection by five viruses (including CMV) from different families indicated that tolerance was effective only against CMV, but not against the other four viruses [90]. This result argues against the hypothesis that tolerance is a general response against different types of stresses [33]. Several experimental works have analyzed how environmental conditions affect the expression of tolerance. Pagán et al. [91] showed that increased *A. thaliana* density reduced tolerance to CMV when the surrounding plants were not infected. Using the same experimental system, Hily et al. [59] provided evidence that increased light and medium temperatures boosted plant point tolerance to infection. Results of these studies would be compatible with the hypothesis that higher resource availability promotes the appearance of tolerance [31]. On the other hand, mortality tolerance to infection by several viruses has been shown to be higher under drought conditions than under high water availability [92]. Analyses using CMV and *A. thaliana* have shown that long-lived host genotypes are more tolerant than short-lived ones, in accordance with the Herms & Mattson [31] model [66,93]. Lastly, Vijayan et al. [67] analyzed the evolution of *A. thaliana* and *Brassica juncea* tolerance to *Turnip mosaic virus* (TMV), using mortality (as expected time until death from infection) as a measure of plant fitness. Results showed genetic variation for this trait among host species. Serial passages of TuMV in *A. thaliana* resulted in reduced plant mortality and reduced resistance, in agreement with models predicting trade-offs between tolerance and resistance.

### 4.3. Bacteria

Tolerance to bacteria in an ecological/evolutionary context has been comparatively less studied than for other plant pathogens. Early evidence of point tolerance to bacteria was published in the 1990s based on the *A. thaliana*-*Xhantomonas campestris* pv. *campestris* system [94,95]. However, most work on tolerance to bacterial infection is derived from the *A. thaliana*-*Pseudomonas* spp. system, as several *Pseudomonas* species have been shown to naturally infect *A. thaliana* [96]. For instance, Kover & Schaal [97] analyzed variation in resistance and range tolerance of 19 *A. thaliana* accessions to *P. syringae*. Their results suggested that there are genetic polymorphisms for tolerance and showed that resistance and tolerance to bacteria coexist in the same *A. thaliana* genotype. Goss & Bergelson [70,98] obtained similar results by using the *A. thaliana-P. viridiflava* interaction.

### 4.4. Parasitic Plants

Similar to bacteria, few experimental analyses of the evolution of tolerance to parasitic plants have been done (Table 1). Still, Medel [99] analyzed tolerance of *Echinopsis chilensis* to *Tristerix aphyllus*, finding genetic variation in this trait in the host. In addition, the study did not find a significant selection correlation coefficient between resistance and tolerance, indicating that both defense strategies were not mutually exclusive in *T. aphyllus*. Koskella et al. [100] also found genetic variation in the tolerance of *Urtica dioica* to *Cuscuta europea* and provided evidence of a tolerance cost in terms of host reproductive success, which would help to maintain genetic variation in tolerance, in agreement with the Restif & Koella [19,37] models. Also, Koskella et al. [100] observed that tolerance depended on the plant sex, which is compatible with the Cousineau & Alizon [52] model. Studies in crops have found similar patterns. For instance, Rowntree et al. [101] found genetic variation for point tolerance of barley to the parasitic plants *Rhinanthus minor* and *Rhinanthus angustifolius*. In this system, resistance and tolerance also coexisted in the host.

## 5. Mechanistic Basis and Genetic Determinants of Plant Tolerance to Pathogens

From the experimental work summarized in the previous section, it can be concluded that tolerance is an efficient and widespread defense strategy of plants against many pathogens. The potential of tolerance in plant disease control has prompted the study of its mechanisms and inheritance. Many studies on this subject have been carried out in the context of its potential use for plant breeding. Most of these studies defined tolerance as the reduction in the severity of the symptoms induced by pathogen infection, a trait that in an agricultural context may be more relevant than plant fitness. However, in the plant breeding literature, tolerance is frequently used when reduced symptom severity is accompanied by lower pathogen multiplication, or even when pathogen multiplication is not measured. Because this is against the common concept of tolerance used by both pathologists and evolutionary biologists, we will not further discuss these works. QTLs for tolerance have been identified in several plant-pathogen interactions (e.g., [87,88,89,102,103]). Interestingly, a common conclusion of these works is that a few genes determine tolerance, although their functions have not been characterized. Studies of the mechanisms of tolerance indicate three groups that are not mutually exclusive:

First is tolerance through compensation of the loss of photosynthetic activity due to infection. It has been well established that pathogen infection often inhibits photosynthesis and results in lower carbon fixation, both at the single leaf and at the whole plant scales [104]. In several plant-pathogen interactions, it has been shown that plants can compensate for reduced CO_2_ fixation in infected tissues by increasing photosynthesis in healthy parts of infected leaves and/or in uninfected leaves. For instance, tolerance of *S. vulgaris* to the fungi *C. tussilginis* and *P. lagenophorae* has been shown to be associated with higher CO_2_ fixation at the whole plant scale [76]. Also, Scholes et al. [105] showed that the presence of green functionally photosynthetic areas in oat leaves infected by *Puccinia coronata* could compensate for the loss of photosynthetic activity in other parts of the same leaf damaged by the pathogen. However, whether this compensation was associated with tolerance was not analyzed. Potato (*Solanum tuberosum*) tolerance to *Potato virus Y* (PVY) has been also shown to be linked to modifications of photosynthetic activity [106]: infection by PVY resulted in an early activation of the potato photosynthetic apparatus and in a constant up-regulation of some RuBisCO transcripts. Lastly, this mechanism of tolerance has been reported in faba bean (*Vicia faba*) against the parasitic plant *Orobanche foetida* [107]; tolerant faba bean genotypes suffered a reduction in the content of nitrogen compounds but maintained carbon levels. Thus, the plant photosynthetic machinery may play a relevant role in plant tolerance against different types of pathogens.

Second, tolerance through alteration of the plant developmental schedule to divert resources from growth into reproduction. Based on the concept that trade-offs exist between resources allocated to different fitness components (reproduction, growth and survival), Life-history Theory predicts that the optimal pattern of resource allocation may differ depending on environmental conditions, which include parasitism [108]. Models based on this theory predict that parasitized hosts will allocate more resources to reproduction, subtracting them from those dedicated to growth and survival [11,109]. Life-history theory also states that environmental conditions affecting mortality rates modify temporal life-history schedules in order to maximize fitness [108]. Accordingly, highly virulent pathogens will induce shorter host pre-reproductive periods in order to produce progeny before resource depletion, castration or death. In contrast, low virulence will result in a delay in host reproduction, which allows for compensation of pathogen damage [110]. In agreement with these theoretical predictions, tolerance to CMV in *A. thaliana* has been shown to be associated with resource reallocation from growth into reproduction [65,66,70]. In some *A. thaliana* genotypes, this response was strong enough to result in over-compensation; a phenomenon also reported by Hily et al. [59]. Interestingly, such resource reallocation was not observed in response to infection by other more virulent viruses [90], which suggests that resource reallocation is virus-specific or that it is not effective against other more virulent pathogens. In this regard, studies of the *A. thaliana*-TuMV interaction indicate that tolerance to this highly virulent virus is attained through modifications of the temporal developmental schedule, specifically, shortening of the pre-reproductive period resulted in larger seed production [67]. Also, tolerance of *A. thaliana* to *P. viridiflava* was associated with shorter host pre-reproductive periods [98]. Similarly, tolerance of *A. thaliana* to *H. arabidopsidis* was associated with accelerated plant bolting [79]. All these reports suggest that genes controlling plant flowering may be involved in tolerance through alteration of life-history traits. However, this hypothesis has not been analyzed to date.

Third is tolerance through modification of phytohormone balance in response to infection. Studies on plant-pathogen interactions indicate that the processes of disease symptom development and pathogen growth can be uncoupled [111]. Thus, in many instances, the symptoms associated with disease represent an active host response to the presence of a pathogen but do not influence pathogen growth. These host responses are frequently mediated by the same phytohormones involved in resistance, which in certain host-pathogen interactions may also induce tolerance. For example, ethylene and salicylic acid (SA) mediate symptom development, but not growth, of bacterial pathogens [112,113]. Thus, in tomato (*Solanum lycopersicum*) the expression of ethylene and SA mediates resistance against primary infection with *X. campestris,* as well as tolerance to a secondary challenge by a different strain of the same bacteria [114]. Activation of SA also plays a role in tolerance of *A. thaliana* to *P. syringae* [115]. Analyses by Li et al. [116] also suggested that SA and jasmonic acid could be involved in tolerance of tomato to TYLCV.

## 6. Conclusions and Future Perspective

Resistance is the most exhaustively studied defense process of plants to pathogens. However, this review should make clear that there is a considerable interest in plant tolerance to pathogens, which has resulted in a large body of theory. The amount of experimental or mechanistic analyses is much smaller, and experimental evidence is not always supportive of theoretical predictions. From the existing literature, several common patterns arise: (i) In most analyzed plant-pathogen systems, resistance and tolerance coexist, which indicates that defenses are generally not mutually exclusive, and suggests that tolerance might be as frequent as resistance as a defense strategy; (ii) Despite theory predicting the fixation of tolerance alleles in the host population, evidence of tolerance polymorphisms is abundant regardless of the pathogen considered; (iii) Tolerance is an efficient strategy to reduce the negative effect of infection on host fitness, and in some cases may even lead to over-compensation (i.e., infected plants have higher fitness than uninfected individuals); (iv) Although it has been proposed that increasing tolerance favors higher pathogen multiplication, this association has seldom been explored, and the few published reports did not find evidence of a positive association between plant and pathogen fitness.

Taken together, studies on plant tolerance to pathogens have shown that this defense strategy can provide an interesting approach for the control of plant diseases, and that it has a relevant role in the ecology and evolution of plant-pathogen interactions. Still, some questions remain open that will make an exciting avenue for future research. (i) Despite that a significant fraction of mathematical models on the evolution of tolerance assume that this trait has a cost for the plant, few analyses have experimentally quantified such costs. Because tolerance costs have been proposed as a factor necessary for the coexistence of tolerance and resistance, unveiling tolerance costs and their mechanistic bases will be relevant to understand evolution and coexistence in plant populations exhibiting resistance and tolerance; (ii) In most analyses, tolerance is quantified either as fecundity tolerance or as mortality tolerance. However, it is not clear under which conditions each of these measurements separately fully captures the effect of tolerance on the host and the pathogen fitness, or when both should be quantified. For instance, when the pathogen is only horizontally transmitted, measuring plant mortality could be enough to quantify tolerance, whereas if the pathogen is both horizontally and vertically transmitted, both mortality and fecundity tolerance should be quantified; (iii) Some theoretical elaborations on the evolution of tolerance proposed tolerance-resistance trade-offs, which have been experimentally tested in a few plant-pathogen interactions, with contrasting results. However, it could be possible that tolerance-tolerance trade-offs do also exist if, for instance, higher tolerance to a given pathogen could be traded against tolerance to other pathogens. To date, such tolerance-tolerance trade-offs have not been analyzed; (iv) Only a few experimental analyses have explored the consequences of plant tolerance for the pathogen. Thus, the effect of tolerance on pathogen prevalence, as well as on virulence, i.e., traits that have been proposed to be affected by tolerance, should be analyzed. Addressing these questions would help to achieve a broader view of the evolutionary dynamics of plant tolerance to pathogens and, hence, on the durable use of tolerance for plant disease control.

## Figures and Tables

**Table 1:** Studies of plant tolerance, measured either as range tolerance (RT) or point tolerance (PT), to fungi, oomycetes, viruses, bacteria and parasitic plants.

Tolerance	Pathogen	Species	Factor	Host	Fitness	Reference
RT	Fungus	*Puccinia coronata*	Host genotype	*Avena sativa*	Kernel weight	Polotowski & Browning, 1978 [71]
	Fungus	*Puccinia recondita*	Host genotype	*Triticum aestivum*	Grain production	Roberts et al., 1984 [72]
	Fungus	*Colletotricum dematium*	Pathogen isolate; Host population site	*Ipomea purpurea*	Flower production	Simms & Triplett, 1994 [56]
	Fungus	*Coleosporium tussilginis*	Pathogen isolate	*Senecio vulgaris*	Reproductive biomass	Inglese & Paul, 2006 [76]
	Fungus	*Puccinia lagenophorae*	Pathogen isolate	*Senecio vulgaris*	Reproductive biomass	Inglese & Paul, 2006 [76]
	Oomycete	*Hyaloperonospora arabidopsidis*	Host genotype; Pathogen genotype	*Arabidopsis thaliana*	Seed production	Salvaudon et al., 2007 [77]; 2008 [78]
	Virus	*Cucumber mosaic virus*	Host genotype	*Mimulus gutattus*	Flower production	Carr et al., 2006 [89]
	Virus	*Turnip mosaic virus*	Host species; Pathogen genotype	*Arabidopsis thaliana*	Lifespan	Vijayan et al., 2017 [67]
	Bacteria	*Pseudomoas syringae*	Host genotype	*Arabidopsis thaliana*	Seed production	Kover & Schaal 2002 [97]
	Bacteria	*Pseudomoas viridiflava*	Host genotype; Pathogen isolate	*Arabidopsis thaliana*	Seed production % Mortality	Jakob et al., 2002 [96]; Goss & Bergelson, 2007 [70]
	Plant	Tristerix aphyllus	Infection status	*Echinopsis chilensis*	Branching	Medel 2001 [99]
	Plant	*Cuscuta europea*	Host family; Sex of host plant	*Urtica dioica*	Reproductive biomass	Koskela et al., 2002 [100]
PT	Fungus	*Erysiphe fischeri*	Host genotype	*Senecio vulgaris*	Seed production	Ben-Kalio & Clarke, 1979 [74]
	Fungus	*Puccinia spp.*	Host genotype; Pathogen genotype	Various hosts	Seed production Mortality	Summarized in: Roy & Kirchner, 2000 (Table 2) [16]
	Fungus	*Uromyces spp.*	Host genotype; Pathogen genotype	Various hosts	Seed production Mortality	Summarized in: Roy & Kirchner, 2000 (Table 2) [16]
	Oomycete	*Hyaloperonospora arabidopsidis*	Host family	*Arabidopsis thaliana*	Seed production	Salvaudon & Shykoff, 2013 [79]
	Virus	*Cucumber mosaic virus*	Host inbreeding level	*Mimulus gutattus*	Flower production	Carr et al., 2003 [58]
	Virus	*Cucumber mosaic virus*	Host genotype; Host allometric group; Host density; Pathogen isolate	*Arabidopsis thaliana*	Seed production	Pagán et al., 2007 [65]; 2008 [66]; 2009 [91]
		*Cucumber mosaic virus*	Host genotype; Host allometric group	*Arabidopsis thaliana*	Seed production Lifespan	Hily et al., 2016 [59]
	Bacteria	*Xhantomonas campestris*	Host genotype	*Arabidopsis thaliana*	Chlorophyll content	Tsuji et al., 1991 [94]
	Bacteria	*Xhantomonas campestris*	Host genotype	*Arabidopsis thaliana*	Symptoms	Buell & Somerville, 1995 [95]
	Plant	*Rhinanthus minor*	Host genotype; Pathogen population	*Hordeum vulgare*	Seed production	Rowntree et al., 2011 [101]
		*Rhinanthus angustifolius*	Host genotype; Pathogen population	*Hordeum vulgare*	Seed production	Rowntree et al., 2011 [101]
